# Supplementary material for: A Novel Prognostic Prediction Model Based on Pyroptosis-Related Clusters for Breast Cancer
Source: J Pers Med. 2022 Dec 28;13(1):69. doi: 10.3390/jpm13010069 (PMC9865451; doi:10.3390/jpm13010069)
Supplement: Supplementary file 1 [file jpm-13-00069-s001.zip › jpm-2090227-supplementary.pdf]

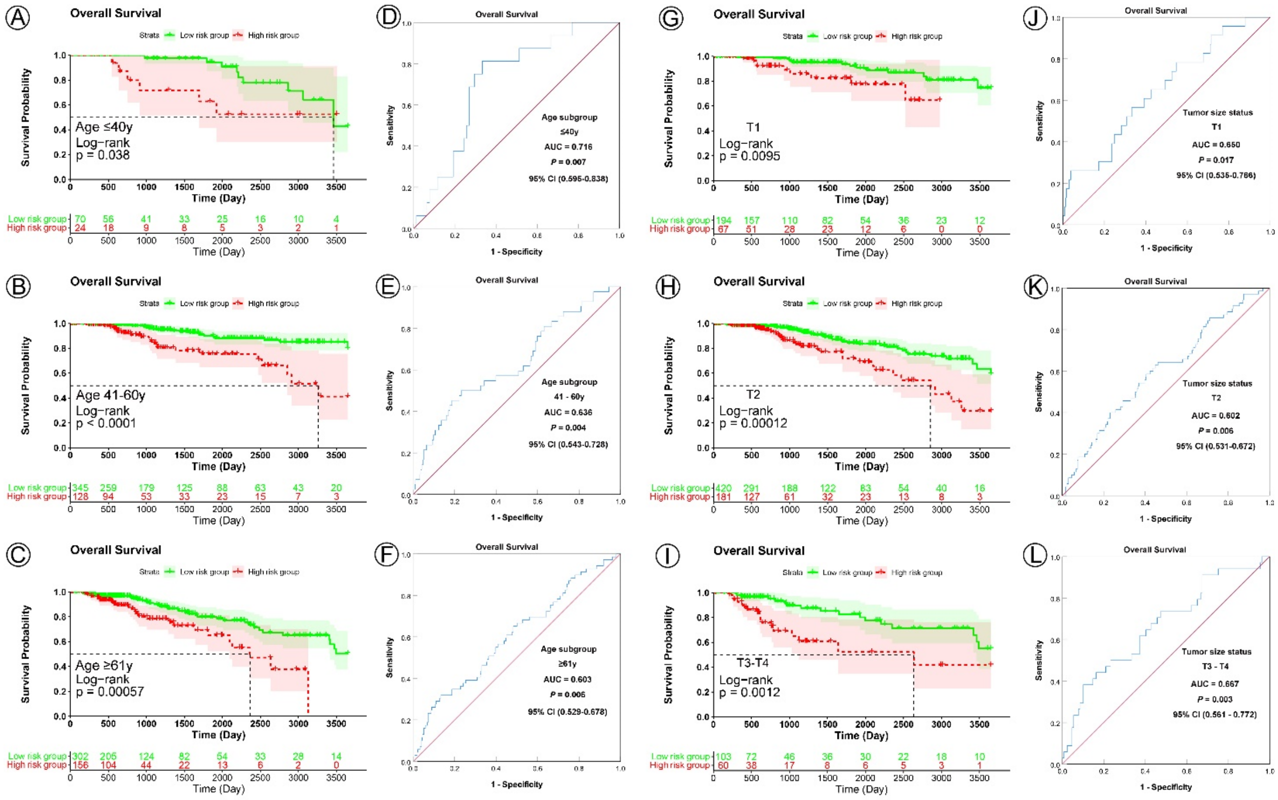

Figure S1. KM and ROC curve analyses of patients stratified by age and tumor size status. (A-C), KM curves of OS for high- and low-risk groups in the ≤ 40-year, 41-60-year, and ≥ 61-year subgroups. (D-F), ROC analysis showed the sensitivity and specificity of prognostic signature for predicting OS for high- and low-risk groups in the ≤ 40-year, 41-60-year, and ≥ 61-year subgroups. (G-I), KM curves of OS for high- and low-risk groups in the T1, T2, and T3-T4 subgroups. (J-L), ROC analysis showed the sensitivity and specificity of prognostic signature for predicting OS for high- and low-risk groups in the T1, T2, and T3-T4 subgroups.

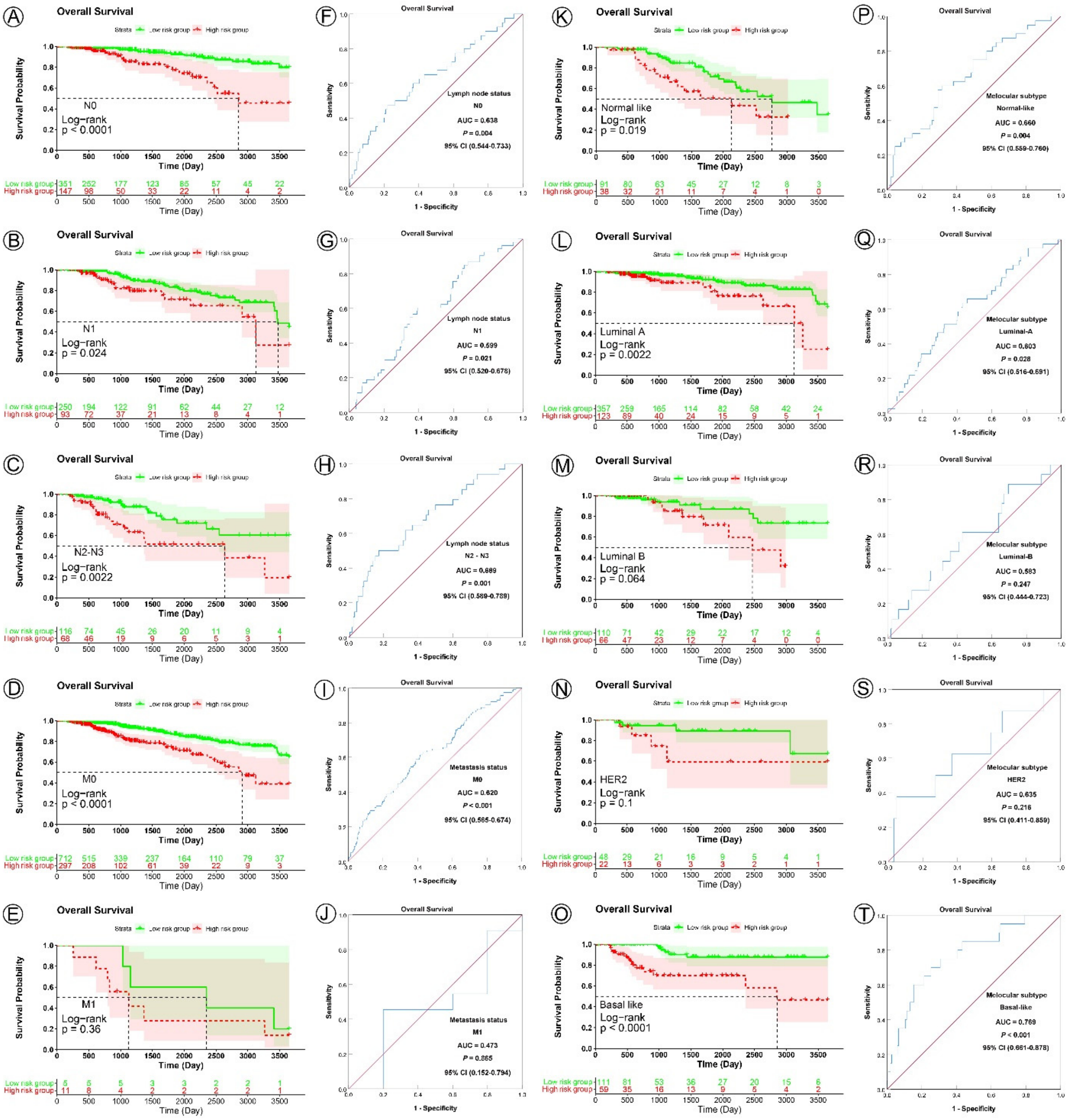

Figure S2. KM and ROC curve analyses of patients stratified by lymph node status, metastasis status, and molecular subtype (PAM50). (A–E), KM curves of OS for high- and low-risk groups in the N0, N1, N2-N3, M0, and M1 subgroups. (F–J) ROC analysis showed the sensitivity and specificity of the prognostic signature for predicting OS for high- and low-risk groups in the N0, N1, N2-N3, M0, and M1 subgroups. (K–O), KM curves of OS for high- and low-risk groups in the normal-like, Luminal A, Luminal B, HER2, Basal-like subgroups. (P–T) ROC analysis showed the sensitivity and specificity of the prognostic gene signature for predicting OS for high- and low-risk groups in the normal-like, Luminal A, Luminal B, HER2, Basal-like subgroup.
